# Associations of objectively measured physical activity, sedentary time and cardiorespiratory fitness with adipose tissue insulin resistance and ectopic fat

**DOI:** 10.1038/s41366-023-01350-0

**Published:** 2023-07-25

**Authors:** Sundus Malaikah, Scott A. Willis, Joseph Henson, Jack A. Sargeant, Thomas Yates, Alice E. Thackray, Fernanda R. Goltz, Matthew J. Roberts, Danielle H. Bodicoat, Guruprasad P. Aithal, David J. Stensel, James A. King

**Affiliations:** 1 National Centre for Sport and Exercise Medicine, School of Sport, Exercise and Health Sciences, Loughborough University, Loughborough, UK; 2 NIHR Leicester Biomedical Research Centre, University Hospitals of Leicester NHS Trust and University of Leicester, Leicester, UK; 3 Clinical Nutrition Department, Faculty of Applied Medical Sciences, King Abdulaziz University, Jeddah, Saudi Arabia; 4 Diabetes Research Centre, University of Leicester, Leicester, UK; 5 Simplified Data, Leicester, UK; 6 Nottingham Digestive Diseases Centre, School of Medicine, University of Nottingham, Nottingham, UK; 7 NIHR Nottingham Biomedical Research Centre, Nottingham University Hospitals NHS Trust and the University of Nottingham, Nottingham, UK; 8 Faculty of Sport Sciences, Waseda University, Tokorozawa, Japan; 9 Department of Sport Science and Physical Education, The Chinese University of Hong Kong, Central Ave, Hong Kong

**Keywords:** Obesity, Pre-diabetes

## Abstract

**Background/objectives:**

Inadequate movement, excess adiposity, and insulin resistance augment cardiometabolic risk. This study examined the associations of objectively measured moderate-to-vigorous intensity physical activity (MVPA), sedentary time and cardiorespiratory fitness (CRF), with adipose tissue insulin resistance and ectopic fat.

**Methods:**

Data were combined from two previous experimental studies with community volunteers (*n* = 141, male = 60%, median (interquartile range) age = 37 (19) years, body mass index (BMI) = 26.1 (6.3) kg·m^-2^). Adipose tissue insulin resistance was assessed using the adipose tissue insulin resistance index (Adipo-IR); whilst magnetic resonance imaging (MRI) was used to measure liver, visceral (VAT) and subcutaneous abdominal adipose tissue (ScAT). Sedentary time and MVPA were measured via an ActiGraph GT3X+ accelerometer. Generalized linear models examined the association of CRF, MVPA, and sedentary time with Adipo-IR and fat depots. Interaction terms explored the moderating influence of age, sex, BMI and CRF.

**Results:**

After controlling for BMI and cardiometabolic variables, sedentary time was positively associated with Adipo-IR (β = 0.68 AU [95%CI = 0.27 to 1.10], *P* < 0.001). The association between sedentary time and Adipo-IR was moderated by age, CRF and BMI; such that it was stronger in individuals who were older, had lower CRF and had a higher BMI. Sedentary time was also positively associated with VAT (β = 0.05 L [95%CI = 0.01 to 0.08], *P* = 0.005) with the relationship being stronger in females than males. CRF was inversely associated with VAT (β = −0.02 L [95%CI = −0.04 to −0.01], *P* = 0.003) and ScAT (β = −0.10 L [95%CI = −0.13 to −0.06], *P* < 0.001); with sex and BMI moderating the strength of associations with VAT and ScAT, respectively.

**Conclusions:**

Sedentary time is positively associated with adipose tissue insulin resistance which regulates lipogenesis and lipolysis. CRF is independently related to central fat storage which is a key risk factor for cardiometabolic disease.

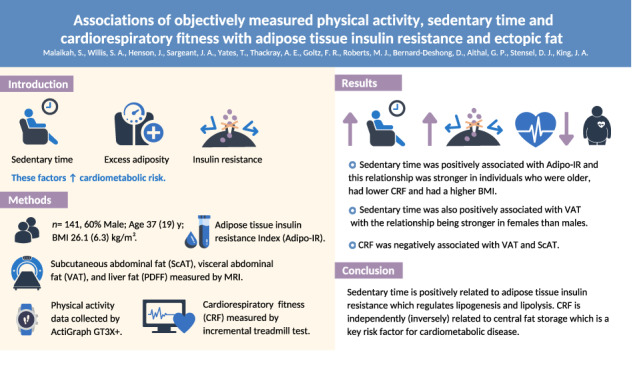

## Introduction

Physical inactivity is an important contributor to the chronic positive energy balance underpinning obesity. Excess adiposity is primarily stored in subcutaneous adipose tissue depots; however, lipid spillover into visceral and ectopic sites occurs once subcutaneous capacity is exceeded [1]. Such regional distribution of fat deposition is an important determinant of associated cardiometabolic risk [2]. Specifically, central fat accumulation, particularly visceral adipose tissue (VAT) and liver fat, are associated with metabolic dysfunction and an augmented risk of several obesity-related diseases [3, 4].

Insulin resistance is a key mediator of the heightened cardiometabolic risk associated with central adiposity [5]. Specifically, elevated VAT and liver fat are both independently associated with impaired insulin sensitivity in the liver, skeletal muscle, and adipose tissue [6, 7]. Adipose tissue insulin resistance may be particularly important in promoting ectopic fat deposition as the reduced ability of insulin to suppress adipose tissue lipolysis, or prompt lipogenesis, leads to the increased delivery of non-esterified fatty acids (NEFA) to ectopic sites [8]. The centrality of this metabolic process to the pathology of obesity-related disease has generated interest around adipose tissue insulin resistance as a target for therapeutic intervention [9, 10].

Regular physical activity, and limiting time spent sedentary, are linked to numerous health benefits including improved insulin sensitivity and reduced central adiposity [11, 12]. Indeed, lower habitual sedentary time, higher moderate-to-vigorous physical activity (MVPA), and greater cardiorespiratory fitness (CRF) have been linked to more favourable whole-body insulin sensitivity and lower subcutaneous abdominal adipose tissue (ScAT), VAT, and liver fat [13–16]. It is unclear, however, if these relationships are modified by important factors such as age, sex, total adiposity and CRF. Furthermore, some [13, 14] but not all [17–19] studies suggest MVPA, sedentary time and CRF are independently associated with cardiometabolic health. Therefore, additional research is required to address these pertinent questions.

To date, no existing studies have examined the relationship of MVPA, sedentary time and CRF with adipose tissue insulin resistance. This is largely due to the lack of specialist expertise and high costs associated with the gold-standard assessment of adipose tissue insulin resistance in vivo using hyperinsulinemic-euglycemic clamp and tracer techniques [20]. However, the adipose tissue insulin resistance index (Adipo-IR) is a simple and inexpensive surrogate marker calculated from basal NEFA and insulin concentrations which correlates closely with clamp-derived measures and in vitro adipocyte insulin action [20, 21]. An accumulating body of evidence has demonstrated that the Adipo-IR is associated with several metabolic derangements including pancreatic beta cell dysfunction [10], dysglycaemia/type 2 diabetes (T2DM) [22], liver inflammation and fibrosis [9, 23]. Such evidence has prompted the suggestion that the Adipo-IR is a biomarker of adipose tissue dysfunction, which is more prognostic for cardiometabolic health than adiposity per se. Although formal MVPA interventions have been shown to improve adipose tissue insulin sensitivity [24, 25], further studies are needed to investigate associations with CRF and sedentary time; and to explore the moderating effect of key demographic and anthropometric factors.

Using baseline data from previous experimental trials, this study examined the associations of objectively measured MVPA, sedentary time and CRF with Adipo-IR and central fat depots determined by magnetic resonance imaging (MRI). As a secondary aim, we explored whether these associations were moderated by key demographic and physiological variables (i.e., age, sex, and body mass index (BMI)). It was hypothesised that Adipo-IR and central fat depots would be positively associated with sedentary time and inversely associated with MVPA and CRF. Furthermore, these associations would be stronger in those who were male, older, and possessing a higher BMI.

## Methods

### Ethical approval

We performed a pooled analysis of data collected across two separate experimental studies; a cross-sectional study [26] and an acute cross-over study [27] conducted at Loughborough University. Both studies were conducted in the same laboratory, used identical protocols/ Standard Operating Procedures, and analytical approaches. Furthermore, both studies obtained ethical approval from Loughborough University’s Human Participants Ethics Advisory Committee. Written informed consent was obtained from all participants before partaking in these studies.

### Participants

Volunteers were recruited from the general population within Leicestershire (UK) by word of mouth, poster, and email advertisement. Data were available for 141 individuals (85 men, 56 women) who were of white European or South Asian ethnicity. Participants were free of T2DM (HbA1c < 48 mmol/mol) and established cardiovascular disease, were weight stable (≤ 3 kg weight change in the last 3 months), non-smokers and were not taking medications known to influence study outcomes. Premenopausal women were not pregnant (self-reported), and tests were completed during the follicular phase of the menstrual cycle.

### Study procedures

The data included in this study were collected during pre-assessment visits and baseline venous blood samples taken at the beginning of experimental trials. Participants refrained from caffeine, alcohol, and structured exercise in the 24 h before laboratory visits. Data collection occurred between November 2016 and September 2019. For each outcome included in these analyses, identical research techniques and biochemical assays were used. The exception was the assessment of CRF (peak oxygen uptake [V̇O_2_ peak]) which was measured directly in 111 participants, whereas it was indirectly determined (Bruce test [28]) in 30 individuals.

### Anthropometry

Height and weight were measured using an integrated stadiometer and digital scale (Seca Ltd, Hamburg, Germany), and BMI was subsequently calculated.

### MRI-derived fat depots

Participants underwent an MRI scan in the supine position to quantify ScAT, VAT, and liver fat content. Image analysis was performed using the AMRA^TM^ profiler (Advanced MR analytics, Sweden) [29]. The scan used a dual-echo Dixon fat and water sequence on a 3T MRI scanner (MR750w, GE Healthcare, Chicago, USA). Seven overlapping image stacks spanning the neck to the knee were acquired during the MRI sequence, with stacks 2 to 5 acquired during breath-hold (femoral head and top of vertebra T9) and used for abdominal fat volume analyses. The IDEAL-IQ sequence was used to assess proton density fat fraction in the liver [30]. After collection, anonymised scans were analysed by AMRA Medical using their AMRA^TM^ profiler (AMRA Medical AB, Linkoping, Sweden).

### Objectively measured physical activity and sedentary time

Participants’ habitual physical activity and sedentary time was assessed over 7-days by use of an ActiGraph GT3X+ accelerometer worn on the waist (ActiGraph, Pensacola, USA). Data were analysed over 15 s epochs (ActiLife, Actigraph corporation, Florida, USA) and classified as follows: <100 counts per minutes as sedentary time, ≥100 to <1952 counts per minute as light physical activity and ≥1952 counts per minute as MVPA [31, 32]. Non-wear time was defined as a minimum of 60 min of continuous zero counts. For inclusion in the analysis, participants were required to provide at least four valid days of measurement, defined as ≥600 min of wear time. Physical activity data are presented per 30 min of activity for ease of interpretation and were used in this study as independent variables and wear time was subsequently entered as a covariate in all statistical models.

### Cardiorespiratory fitness

Within the present study, CRF was determined as the participants’ V̇O_2_ peak and was measured directly via indirect calorimetry for 111 individuals. Conversely, owing to their higher cardiovascular risk, the Bruce test [28] was employed to obtain an indirect measure of V̇O_2_ peak for 30 individuals. Exercise tests were undertaken on a motorised treadmill (Excitemed, Technogym, Cesena, Italy) after familiarisation and a warm-up. Heart rate (Polar T31; Polar Electro, Kempele, Finland) and perceived exertion [33] were measured continuously throughout the tests.

For the direct assessments of V̇O_2_ peak, expired air was sampled continuously during an incremental test to volitional exhaustion, using breath-by-breath analysis (Metalyzer 3B, Cortex, Leipzig, Germany). V̇O_2_ peak was determined as the highest oxygen consumption value averaged over 20 s.

The Bruce test is a progressive test composed of 3 min stages through which treadmill speed and gradient are gradually increased [28]. The test is primarily walking based and is more suitable for individuals with mobility limitation or elevated cardiovascular risk. Test duration, until volitional exhaustion, is entered into a formula to predict V̇O_2_ peak. This indirect measure of V̇O_2_ peak correlates strongly (*r* = 0.97) with that measured directly [28, 34].

### Blood sampling

Venous blood samples were collected from an antecubital vein after participants had fasted overnight. Participants lay in a semi-supine position for 5 min before samples were taken. The blood samples were drawn into pre-cooled EDTA monovettes (Sarstedt, Leicester, United Kingdom) and spun immediately in a refrigerated (4 °C) centrifuge (Labofuge 400 R, ThermoScientific, Langenselbold, Germany) at 1500 x *g* for 10 min. The plasma supernatant was subsequently aliquoted and stored at −80 °C prior to batch analysis.

### Biochemical analysis

Plasma concentrations of high-density lipoprotein (HDL), triacylglycerol (TAG), glucose (Horiba Medical, Montpellier, France) and NEFA (Randox Laboratories Ltd, County Antrim, UK) were spectrophotometrically determined using commercially available kits and a benchtop analyser (Pentra 400, Horiba Medical, Montpellier, France). Plasma insulin was measured via an enzyme-linked immunosorbent assay (Mercodia, Uppsala, Sweden). The coefficient of variance for HDL, TAG, NEFA, glucose and insulin were 0.70%, 0.97%, 1.57%, 0.55%, 3.85%, respectively. The adipose tissue insulin resistance index (Adipo-IR) was calculated as fasting plasma NEFA multiplied by fasting plasma insulin [35]. Adipo-IR is a surrogate marker of adipose tissue insulin resistance which has been validated against clamp-derived measures and in vitro adipocyte insulin responsiveness [21, 35].

### Statistical analyses

Statistical analyses were performed using SPSS version 24 (SPSS Inc., Chicago, Illinois). Kolmogorov-Smirnov tests were used to assess the distribution of the data. Participant characteristics are presented as mean±SD for normally distributed data, median (interquartile range) for non-normally distributed data, and number (percentage) for categorical data. Independent-sample *t*-tests and Mann-Whitney U tests (for non-normally distributed data) were used to assess differences in participant characteristics between individuals with and without missing data. The independent associations of physical activity (MVPA and sedentary time) and CRF with Adipo-IR and MRI-derived fat depots (liver fat, VAT and ScAT) were assessed using generalized linear models. Due to the right-skewed distributions of these variables, a gamma distribution with an identity link function was used. Model 1 was adjusted for demographic variables including study, age (continuous), sex (men/women), and ethnicity (white European/South Asian) plus device wear time (continuous) where physical activity variables were included in the model. Model 2 was additionally adjusted for cardiometabolic variables, namely BMI, glucose, TAG, and HDL (all continuous). Model 3 was further adjusted for CRF, sedentary time, and/or MVPA (all continuous). Multicollinearity between covariates was assessed for each model, thus light physical activity was not included in the analyses due to multicollinearity with sedentary time. Significant associations in model 3 were then explored further by simultaneously adding interaction terms into the models to assess whether these associations were modified by sex, age and BMI. In addition, because interventions to reduce sedentary behaviour have been shown to be more effective at improving metabolic health in those with lower fitness [36], we further assessed interactions between CRF and physical activity variables. To facilitate interpretation, interactions between continuous variables were also stratified using the median split. To caution against type 1 error in our generalized linear models, we performed a sensitivity analysis whereby the alpha level was adjusted using the Holm-Bonferroni sequential procedure [37]. All data for the regression analyses are presented as coefficients (β; 95% confidence intervals). Statistical significance was set at *P* < 0.05 for main effects and *P* < 0.10 for interactions (given that interaction tests have lower power).

## Results

The characteristics of included participants are shown in Table 1. Due to technical issues with the accelerometer and contraindications to the MRI procedures, physical activity, VAT and ScAT data are presented for *n* = 130. Additionally, liver fat could not be determined in a further four participants due to motion artefacts, thus these data are presented as *n* = 126. Apart from ethnicity (*P* = 0.025), there were no statistically significant differences in participant characteristics between those without missing data (*P* ≥ 0.133).

**Table 1:** Participant characteristics.

Demographic variables	Combined (*n* = 141)		Female (*n* = 56)		Male (*n* = 85)	
Ethnicity (white European)	119	[84]	51	[91]	68	[80]
Age (years)	37.0	(19.0)	34.5	(14.7)	38.0	(17.0)
Height (cm)	172.8	±8.9	165.2	±6.1	177.8	±6.6
Weight (kg)	80.9	±19.7	66.5	±11.1	90.3	±18.3
*Cardiometabolic variables*						
BMI (kg∙m^-2^)	26.1	(6.3)	24.1	(4.9)	27.4	(6.4)
Glucose (mmol∙L^−^^1^)	5.4	(0.8)	5.0	(4.7)	5.5	(0.8)
Insulin (pmol∙L^−^^1^)	24	(24)	20	(13)	26	(32)
NEFA (mmol∙L^−^^1^)	0.4	(0.2)	0.4	(0.3)	0.4	(0.2)
TAG (mmol∙L^−^^1^)	0.9	(0.6)	0.7	(0.2)	1.1	(0.9)
HDL (mmol∙L^−^^1^)	1.2	± 0.3	1.4	±0.3	1.2	±0.2
Adipo-IR (AU)	10.4	(13.0)	8.5	(8.4)	11.0	(18.1)
*MRI-derived variables*						
Liver fat (%)^a^	1.8	(2.1)	1.3	(0.9)	2.3	(5.8)
VAT (L)^a^	1.8	(2.6)	0.9	(0.7)	2.8	(3.4)
ScAT (L)^a^	5.8	(4.6)	5.7	(4.3)	5.8	(5.1)
*Cardiorespiratory fitness, physical activity, and sedentary time*						
CRF (mL∙kg^−^^1^∙min^−^^1^)	40.8	± 9.8	38.9	± 6.0	42.1	±11.5
Light PA (min∙d^−^^1^)^a^	278	±78	299	±83	265	±73
MVPA (min∙d^−^^1^)^a^	50	(41)	46	(41)	50	(41)
Sedentary time (min∙d^−^^1^)^a^	580	±95	557	±82	595	±100
Device wear time (min∙d^−^^1^)^a^	925	(73)	917	(63)	926	(83)

Data are presented as mean ± SD, median (interquartile range) or number [column percentage]. *Adipo-IR* adipose tissue insulin resistance index, *BMI* body mass index, *CRF* cardiorespiratory fitness, *HDL* high-density lipoprotein, *MRI* magnetic resonance imaging, *MVPA* moderate-to-vigorous physical activity, *NEFA* non-esterified fatty acids, *PA* physical activity, *ScAT* subcutaneous abdominal adipose tissue, *TAG* triacylglycerol, visceral adipose tissue (VAT). ^a^Please note *n* = *130 for VAT, ScAT* and physical activity data and *n* = *126* for liver fat data.

### Sedentary time

Associations of sedentary time with Adipo-IR and MRI-derived fat depots are presented in Table 2. Following adjustment for demographic variables (model 1), sedentary time was positively associated with Adipo-IR (0.68 AU [0.18 to 1.18]). Additionally, after further adjustment for BMI, cardiometabolic variables, CRF and MVPA (models 2 and 3), the positive association between sedentary time and Adipo-IR remained; whilst sedentary time was also positively associated with VAT. These data show that after controlling for relevant covariates, each 30 min of sedentary time was associated with a 0.59 AU (0.01 to 1.08) higher Adipo-IR and 0.04 L (0.00 to 0.08) higher VAT. In a sensitivity analysis involving the sequential adjustment of alpha for multiple comparisons, the associations of sedentary time with Adipo-IR and VAT in model 3 no longer remained significant (Table 2). Supplementary Figure 1 visually displays the associations between sedentary time and the study outcome variables.

**Table 2:** Associations of cardiorespiratory fitness and objectively measured physical activity and sedentary time with Adipo-IR and MRI-derived fat depots.

	*CRF* (mL∙kg^−^^1^∙min^−^^1^)^bc^		Sedentary time (per 30 mins)^ac^		MVPA (per 30 mins)^ab^	
	β (95% CI)	*P-*value	β (95% CI)	*P-*value	β (95% CI)	*P-*value
*Model 1*						
Adipo-IR (AU)	**−0.33 (−0.50 to −0.16)**	**<0.001**	**0.68 (0.18 to 1.18)**	**0.007**	−1.08 (−2.14 to −0.01)	0.049
Liver fat (%)	−**0.07 (**−**0.11 to** −**0.04)**	**<0.001**	0.04 (−0.07 to 0.16)	0.472	−0.15 (−0.40 to 0.09)	0.217
VAT (L)	−**0.06 (**−**0.08 to** −**0.04)**	**<0.001**	0.01 (−0.04 to 0.07)	0.605	−0.05 (−0.19 to 0.08)	0.442
ScAT (L)	−**0.19 (**−**0.24 to** −**0.14)**	**<0.001**	0.07 (−0.12 to 0.25)	0.483	−0.38 (−0.73 to −0.03)	0.034
*Model 2*						
Adipo-IR (AU)	−0.15 (−0.35 to 0.06)	0.154	**0.68 (0.27 to 1.10)**	**<0.001**	−1.13 (−2.04 to −0.21)	0.016
Liver fat (%)	−0.00 (−0.05 to 0.04)	0.873	0.06 (−0.05 to 0.18)	0.282	−0.21 (−0.43 to 0.01)	0.062
VAT (L)	−**0.02 (**−**0.04 to** −**0.01)**	**0.001**	**0.05 (0.01 to 0.08)**	**0.005**	−0.09 (−0.17 to 0.00)	0.054
ScAT (L)	−**0.10 (**−**0.13 to** −**0.07)**	**<0.001**	0.05 (−0.05 to 0.15)	0.311	0.12 (−0.16 to 0.40)	0.416
Model 3						
Adipo-IR (AU)	−0.02 (−0.23 to 0.19)	0.822	0.59 (0.01 to 1.08)	0.019	−0.65 (−1.71 to 0.41)	0.227
Liver fat (%)	−0.01 (−0.05 to 0.04)	0.827	0.01 (−0.12 to 0.15)	0.827	−0.20 (−0.47 to 0.08)	0.156
VAT (L)	−**0.02 (**−**0.04 to** −**0.01)**	**0.003**	0.04 (0.00 to 0.08)	0.048	−0.04 (−0.14 to 0.07)	0.501
ScAT (L)	−**0.10 (**−**0.13 to** −**0.06)**	**<0.001**	−0.01 (−0.10 to 0.08)	0.774	0.13 (−0.14 to 0.39)	0.344

Model 1 adjusted for study, sex, ethnicity, age and device wear time. Model 2 adjusted for all of the previous covariates plus BMI, glucose, TAG and HDL. Model 3 adjusted for all of the previous covariates plus ^a^*CRF*, ^b^*sedentary time* or ^c^*MVPA*. Bold font indicates that associations remained statistically significant after applying Holm-Bonferroni sequential correction for multiple comparisons.

*Adipo-IR* adipose tissue insulin resistance index, *BMI* body mass index, *CRF* cardiorespiratory fitness, *HDL* high-density lipoprotein, *MVPA* moderate-to-vigorous physical activity, *ScAT* subcutaneous abdominal adipose tissue, *TAG* triacylglycerol, *VAT* visceral adipose tissue.

### Moderate-to-vigorous physical activity

Table 2 and Supplementary Fig. 1 show the associations of MVPA with Adipo-IR and MRI-derived fat depots. After adjusting for demographic variables (model 1), every 30 min of MVPA was inversely associated with Adipo-IR (−1.08 AU [−2.14 to −0.01]) and ScAT (−0.38 L [−0.73 to −0.03]). The inverse association with Adipo-IR remained significant following further adjustment for BMI and cardiometabolic variables (model 2) but not when sedentary time and CRF were added as additional covariates (model 3). The inverse association between MVPA and ScAT (model 1) was no longer significant in model 2 when BMI and cardiometabolic variables were added as covariates. In a sensitivity analysis involving the sequential adjustment of alpha for multiple comparisons, the associations of MVPA with Adipo-IR and ScAT were no longer significant in any of the models (Table 2).

### Cardiorespiratory fitness

Associations of CRF with Adipo-IR and MRI-derived fat depots are shown in Table 2 and Supplementary Fig. 1. Following adjustment for demographic variables (model 1), each 1 mL∙kg^−^^1^∙min^−^^1^ increase in CRF was inversely associated with Adipo-IR (−0.33 AU [−0.50 to −0.16]), liver fat (−0.07% [−0.11 to −0.04]), VAT (−0.06 L [−0.08 to −0.04]) and ScAT (−0.19 L [−0.24 to −0.14]). Following further adjustment for BMI, cardiometabolic and physical activity variables (model 3), the inverse association of CRF with VAT (−0.02 L [−0.04 to −0.01]) and ScAT (−0.10 L [−0.13 to −0.06]) remained. Conversely, associations of CRF with Adipo-IR and liver fat identified in model 1 were no longer apparent when BMI and cardiometabolic variables were added as covariates in model 2. In a sensitivity analysis involving the sequential adjustment of alpha for multiple comparisons, the associations of CRF with all aforementioned outcome variables remained statistically significant across models 1 to 3 (Table 2).

### Interaction analyses

Significant interaction analyses with sex, age, BMI and CRF are shown in Table 3. Interaction analyses revealed that sex moderated the inverse association between CRF and VAT which was present in males (−0.04 L [−0.08 to 0.01]) but not females (−0.01 L [−0.03 to 0.01]). Conversely, sedentary time was positively associated with VAT in females (0.06 L [0.02 to 0.10]) but not males (−0.01 L [−0.14 to 0.11]).

**Table 3:** Statistically significant interaction analyses with sex, age, body mass index, cardiorespiratory fitness and objectively measured physical activity and sedentary time.

Outcome	Explanatory variable	*n*	*P-*value for interaction	Category 1 β (95% CI)	Category 2 β (95% CI)
*Sex*				**Male**	**Female**
VAT (L)	*CRF* (mL∙kg^−^^1^∙min^−^^1^)^bc^	121	0.024	−0.04 (−0.08 to 0.01)	−0.01 (−0.03 to 0.01)
VAT (L)	Sedentary time (per 30 min)^ac^	121	0.074	−0.01 (−0.14 to 0.11)	0.06 (0.02 to 0.10)
*Age*				< **37 years**	≥ **37 years**
Adipo-IR (AU)	Sedentary time (per 30 min)^ac^	129	0.041	0.33 (−1.29 to 1.95)	1.09 (0.40 to 1.78)
*BMI*				**<26.1** **kg** ∙ **m**^**-2**^	≥ **26.1** **kg** ∙ **m**^**-2**^
Adipo-IR (AU)	Sedentary time (per 30 min)^ac^	129	0.063	0.23 (−1.24 to 1.70)	1.18 (0.53 to 1.84)
ScAT (L)	*CRF* (mL∙kg^−^^1^∙min^−^^1^)^bc^	121	0.068	−0.10 (−0.28 to 0.09)	−0.24 (−0.33 to −0.15)
*Cardiorespiratory fitness*				<**40.1** **mL∙kg**^-1^**∙min**^**-1**^	≥**40.1** **mL∙kg**^-1^**∙min**^**-1**^
Adipo-IR (AU)	Sedentary time (per 30 min)^*ac*^	129	0.039	1.08 (−0.55 to 2.71)	0.21 (−0.47 to 0.89)

Data are presented as *P*-values for the interaction term and as β-coefficients (95% confidence intervals) for categorical variables and variables stratified using the median split.

Adjusted for study, sex, ethnicity, age, device wear time, BMI, glucose, TAG, HDL, interaction term and ^a^CRF, ^b^sedentary time or ^c^MVPA.

*Adipo-IR* adipose tissue insulin resistance index, *BMI* body mass index, *CRF* cardiorespiratory fitness, *HDL* high-density lipoprotein, *MVPA* moderate-to-vigorous physical activity, *ScAT* subcutaneous abdominal adipose tissue, *TAG* triacylglycerol, *VAT* visceral adipose tissue.

Interaction analyses showed that age moderated the positive association between sedentary time and Adipo-IR. Subsequent stratification demonstrated that this association was evident in older individuals (≥ 37 years) where every 30 min of sedentary time was associated with a 1.09 AU [0.40 to 1.78] higher Adipo-IR, however this relationship was not apparent in individuals aged below 37 years (0.33 AU [−1.29 to 1.95]).

Interaction analyses further revealed that BMI moderated the positive association between sedentary time and Adipo-IR. After stratification, each 30 min of sedentary time was associated with a 1.18 AU (0.53 to 1.84) higher Adipo-IR in those with a higher BMI (≥ 26.1 kg∙m^−^^2^); whereas this association was not present in those with a lower BMI (< 26.1 kg∙m^−^^2^; 0.23 AU [−1.24 to 1.70]). Additionally, BMI moderated the inverse association between CRF and ScAT such that each 1 mL∙kg^−^^1^∙min^−^^1^ increase in CRF was associated with a 0.24 L (−0.33 to −0.15) lower ScAT in those with a higher BMI. In comparison, this association was weaker in those with a lower BMI (−0.10 L [−0.28 to 0.09]).

Interaction analyses for CRF showed that the association between sedentary time and Adipo-IR was modified by CRF such that each 30 min of sedentary time was associated with a 1.08 AU (−0.55 to 2.71) higher Adipo-IR in individuals with a lower CRF (< 40.1 mL∙kg^−^^1^∙min^−^^1^). In contrast, this association was not present in individuals with a higher CRF (≥ 40.1 mL∙kg^−^^1^∙min^−^^1^; 0.21 AU [−0.47 to 0.89]).

## Discussion

This study investigated the association of objectively measured CRF, physical activity and sedentary time with adipose tissue insulin resistance and depot-specific measurements of adiposity in a cohort of adults without established metabolic disease. The novel findings are three-fold. Firstly, habitual sedentary time was positively associated with Adipo-IR and this relationship was stronger in those who were older, had a larger BMI and lower levels of CRF. Secondly, CRF was inversely related to VAT with a stronger relationship observed in men versus women. Thirdly, CRF was inversely associated with ScAT with stronger associations apparent for individuals with a higher BMI.

Adipose tissue insulin resistance is characterised by impaired suppression of lipolysis and stimulation of lipogenesis in the presence of high circulating insulin [10]. With weight gain, the generation of large and inflamed adipocytes attenuates the sensitivity of adipose tissue to insulin; resulting in augmented circulating NEFA concentrations and impaired glucose regulation [38, 39]. These responses are central to the pathophysiology of T2DM and non-alcoholic fatty liver disease (NAFLD) [40]. A hyper-insulinemic euglycemic clamp with lipid (palmitate and/or glycerol) tracer is the gold standard methods for measuring adipose tissue insulin sensitivity; however, this technique is invasive and require significant technical expertise. Fortunately, this outcome can be assessed indirectly using Adipo-IR, an index composed of fasting NEFA and insulin concentrations [20, 21]. Preliminary research examining the impact of exercise training on adipose tissue insulin sensitivity using hyper-insulinemic euglycemic clamp produced mixed findings, with some studies identifying improvements [24, 25] whilst others observed no change [41, 42]. Our results extend this evidence by showing that Adipo-IR is inversely associated with CRF and is positively associated with sedentary time. Only the associations with sedentary time remained after adjustment for cardiometabolic variables. No other studies have directly examined the relationship between sedentary time and measures of adipose tissue insulin resistance.

Some previous research has demonstrated that sedentary behaviour exerts specific effects on gene expression and substrate metabolism in skeletal muscle which may not be overcome by short bouts of structured exercise [43]. This may have subsequent consequences for system-wide metabolic control [44]. However, the degree to which the association with sedentary time was independent of MVPA in the present analysis was uncertain. Follow-up experimental studies are needed to scrutinise changes in adipose tissue lipid metabolism in response to prolonged inactivity.

Another novel outcome in our analyses was that the relationship between sedentary time and Adipo-IR was modified by age, CRF and BMI; such that it was stronger in individuals who were older, less fit, and had a higher BMI. A potential explanation regarding the mediating effect of age and BMI is that greater fat accumulation is associated with larger and older adipocytes which are more insulin resistant and inflamed [45, 46]. Consequently, the detrimental metabolic impact of excess sedentary time may have a more deleterious effect in tissue that is already primed. Moreover, CRF may mediate the relationship by modulating skeletal muscle – adipose tissue cross-talk, or via direct effects within adipose tissue [42, 47].

In our analyses, VAT was positively associated with sedentary time independent of BMI and cardiometabolic variables, but not MVPA after adjustment for multiple testing. Interestingly, both VAT and ScAT were inversely associated with CRF independent of MVPA and sedentary time. The strength of these associations with VAT were moderated by sex, and the association between CRF and ScAT was also moderated by BMI.

The observed relationship between VAT and sedentary time is consistent with two previous studies reporting positive associations between MRI-derived VAT and objectively measured sedentary time in individuals at high risk of T2DM [14, 48]. Notably, Henson et al. [48] found this association was stronger in males when compared to females which contrasts with our finding where the association was stronger in females compared to males. Although this was not what we would expect since there are inherent differences in regional fat distribution between men and women, with men preferentially storing fat intra-abdominally, particularly as VAT [49]. The discrepancy could be due to the men with central obesity in our analysis being more active compared to Henson et al.’s [48] more sedentary population at high risk of T2DM.

Only one previous study has examined the relationship between MRI-derived VAT and objectively measured CRF which reported a similar inverse relationship to the present study [16]. These data corroborate experimental evidence demonstrating that regular exercise training can reduce VAT content independent of weight loss [50]. CRF is determined by both intrinsic factors (genetics) and habitual physical activity levels, particularly of vigorous intensity [51]. The lack of association that we saw between MVPA and VAT may imply that genetic factors contributing to aerobic fitness, and particularly their interaction with MVPA, may have driven the association between CRF and VAT. Nonetheless, the importance of aerobic fitness improvement has important implications for public health as VAT accumulation is associated with a greater cardiometabolic risk [52]. Notably, in contrast to Chartrand et al. [16], we found that sex also moderated this relationship, such that it was stronger in men than women. This discrepancy may be related to differences in age and CRF between the female populations recruited. Specifically, in the study by Chartrand et al. [16], the female participants were, on average, less fit and of menopausal age which is independently associated with accelerated VAT accumulation [53, 54].

In the present study, ScAT was also inversely associated with CRF independent of MVPA and sedentary time. These data support findings from exercise training interventions [55] and suggest that purposeful physical activity, of sufficient intensity to improve CRF, is also linked to lower ScAT accumulation. Given that VAT was also associated with CRF, this finding may be expected as central fat accumulation is characterised by the expansion of both VAT and ScAT depots [1]. Whilst subcutaneous adipose tissue is regarded as a comparatively ‘safer’ fat depot [56], ScAT accumulation still confers a greater cardiometabolic risk compared to other regions [57]. However, this is most likely a reflection of concomitant fat deposition in other central depots such as VAT and the liver. We also observed that the inverse association between ScAT and CRF was stronger in individuals with a higher BMI. As alluded to previously, it would be expected that those with a higher BMI would have a broader range of ScAT and may therefore possess a greater capacity for change with improvements in CRF.

Surprisingly, liver fat was not related to CRF, MVPA or sedentary time in the fully adjusted model. These findings contrast those of other studies which have reported inverse associations with CRF [16, 58] and MVPA [59, 60], and positive associations with sedentary time [14, 48, 59]. Importantly, the participants in these studies were either at high-risk of T2DM and other chronic diseases or had diagnosed NAFLD. Conversely, mean liver fat percentage of the present study cohort was low (1.8%) and most participants were well below the clinical threshold for NAFLD (i.e. ≥ 5.6%) which may have limited our ability to detect associations between these outcomes. Interestingly, similar to the associations between CRF and Adipo-IR, the inverse relationship between CRF and liver fat seen in model 1 disappeared in model 2 when BMI and cardiometabolic variables were adjusted for. This is likely because liver fat and Adipo-IR are related to BMI so that controlling for these variables weakens associations.

The lack of independent association between MVPA and ScAT was another surprise finding in our study. Further analyses (not presented) showed that the lack of association beyond model 1 was related to the adjustment for BMI. Indeed, when BMI was removed as a covariate in model 2 and 3, the beta-coefficient strengthened (became more negative) and was statistically significant in model 2.

A key strength of this study is the objective measurement of movement-related outcomes and precise assessment of fat depots using MRI. The inclusion of a diverse group of community volunteers is also noteworthy. Limitations of these data include the assessment of adipose tissue insulin resistance using Adipo-IR rather than a direct measurement. Furthermore, participants’ dietary habits were not recorded which may have been a potential confounding variable (i.e., as movement behaviours may associate with eating behaviours and/or diet quality). Additionally, the relatively young age of our adult sample may limit external validity, whilst the use of the ActiGraph GT3X+ to measure movement behaviour prevents us knowing the nature of sedentary behaviours (i.e., whether sedentary behaviour was sitting, lying down, or standing still). Finally, the observational nature of our findings should also be recognised as causality cannot be inferred. Relatedly, as an alternative to our current approach, compositional approaches can be considered to examine the influence of exchanging one movement behaviour for another. However, based on the cross-sectional design of the study, compositional approaches were not adopted in the present analyses to avoid overstating findings. Instead, our findings are intended for hypothesis generation to stimulate future research to test these identified relationships in experimental settings.

In conclusion, the present study found that in a sample of community volunteers, sedentary time is positively associated with both Adipo-IR and VAT. Furthermore, CRF is inversely associated with VAT and ScAT. These findings suggest that spending too much time sedentary may be linked to the development of adipose tissue insulin resistance and visceral adiposity. However, low levels of CRF may also be linked to central fat accumulation. Additional studies are now required to determine the causal nature of these associations through experimental studies in clinical groups.

### Supplementary information

Supplementary Materials

## Data Availability

The datasets analysed during the current study are available from the corresponding author on reasonable request.
