# Supplementary material for: Associations of objectively measured physical activity, sedentary time and cardiorespiratory fitness with adipose tissue insulin resistance and ectopic fat
Source: Int J Obes (Lond). 2023 Jul 25;47(10):1000–7. doi: 10.1038/s41366-023-01350-0 (PMC10511317; doi:10.1038/s41366-023-01350-0)
Supplement: Supplementary file 1 — Supplementary Materials [file 41366_2023_1350_MOESM1_ESM.docx]

**Supplementary materials**

**
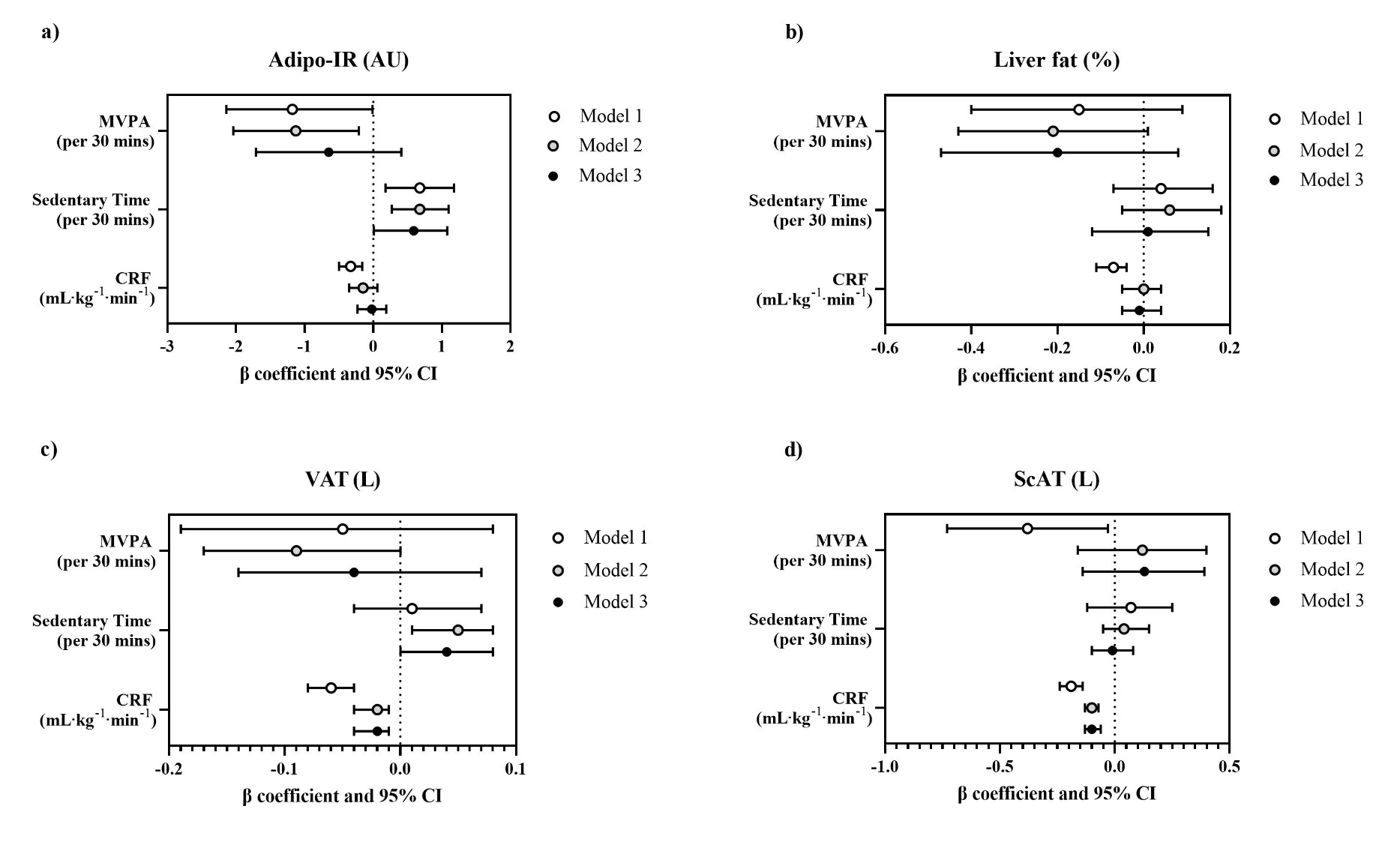
**

**Supplementary Figure 1.** Forest plot showing the associations of CRF, sedentary time, and MVPA with a) Adipo-IR, b) liver fat, c) VAT, and d) ScAT. Values represent β coefficient and 95% CI. Adipo-IR, adipose tissue insulin resistance index; CRF, cardiorespiratory fitness; MVPA, moderate–vigorous intensity physical activity; ScAT, subcutaneous abdominal adipose tissue; VAT, visceral adipose tissue.
